# High-temperature thermal stability driven by magnetization dilution in CoFeB free layers for spin-transfer-torque magnetic random access memory

**DOI:** 10.1038/s41598-018-32641-6

**Published:** 2018-09-26

**Authors:** Jodi M. Iwata-Harms, Guenole Jan, Huanlong Liu, Santiago Serrano-Guisan, Jian Zhu, Luc Thomas, Ru-Ying Tong, Vignesh Sundar, Po-Kang Wang

**Affiliations:** TDK – Headway Technologies, Inc., 463 S. Milpitas Boulevard, Milpitas, CA 95035 USA

## Abstract

Spin-transfer-torque magnetic random access memory (STT-MRAM) is the most promising emerging non-volatile embedded memory. For most applications, a wide range of operating temperatures is required, for example −40 °C to +150 °C for automotive applications. This presents a challenge for STT-MRAM, because the magnetic anisotropy responsible for data retention decreases rapidly with temperature. In order to compensate for the loss of thermal stability at high temperature, the anisotropy of the devices must be increased. This in turn leads to larger write currents at lower temperatures, thus reducing the efficiency of the memory. Despite the importance of high-temperature performance of STT-MRAM for energy efficient design, thorough physical understanding of the key parameters driving its behavior is still lacking. Here we report on CoFeB free layers diluted with state-of-the-art non-magnetic metallic impurities. By varying the impurity material and concentration to modulate the magnetization, we demonstrate that the magnetization is the primary factor driving the temperature dependence of the anisotropy and thermal stability. We use this understanding to develop a simple model allowing for the prediction of thermal stability of STT-MRAM devices from blanket film properties, and find good agreement with direct measurements of patterned devices.

## Introduction

STT-MRAM devices composed of magnetic tunnel junctions (MTJs) exploit the interfacial perpendicular magnetic anisotropy (PMA) that arises between ferromagnetic CoFeB and insulating MgO thereby enabling deep scaling and low switching currents^1–3^. Despite recent advances in STT-MRAM technology^1–10^, energy efficiency remains a challenge because of the wide operating temperature range highlighted in Table 1. The reason for this challenge comes from the combination of two factors. First, the switching current at a given temperature is directly linked to the thermal stability factor ${\rm{\Delta }}=\frac{{E}_{b}}{{k}_{B}T}$, where *E*_*b*_ is the energy barrier between parallel and anti-parallel states, *T* is the absolute temperature and *k*_*B*_ is the Boltzmann constant. Second, memory data retention is determined by Δ, which is strongly dependent on temperature. Indeed, in contrast with traditional silicon-based memories, for which *E*_*b*_ is roughly constant and Δ ~ 1/*T*, *E*_*b*_ of a ferromagnetic free layer decreases with temperature due to the decrease of saturation magnetization *M*_*s*_ and anisotropy field *H*_*k*_. In order to retain thermal stability at high temperatures, *E*_*b*_ must be increased, leading to high switching currents at low temperatures. Thus, achieving high efficiency requires minimizing the temperature dependence of the free layer’s magnetic properties. This is particularly important for applications which require data retention after the reflow soldering process needed for chip packaging. In this case, STT-MRAM devices must maintain data retention at 260 °C, but must still be written at −40 °C. This adds up to an operating range of 300 °C, within which the variations of Δ must be kept as small as possible.

**Table 1:** Temperature requirements for STT-MRAM devices.

Application	Operating Temperatures	Data Retention		Thermal Budget for Manufacturing
		Operation	Solder Reflow	
Commercial	0 to 70 °C	>10 years at the maximum operating temperature	90 seconds at 260 °C	400 °C, up to 5 hours
Industrial	−40 to 85 °C			
Automotive	−40 to 150 °C			
Military	−55 to 125 °C			

In order to gain a deeper understanding of the origin of the temperature variations of Δ, we have investigated the magnetic properties of CoFeB (CFB) free layers diluted with varying amounts of non-magnetic, metallic impurities from groups V-A and VI-A, such as Mo, W, Ta, Nb, etc., which are widely used in state-of-the-art STT-MRAM devices^11–16^. We find that increased magnetization dilution at room temperature also leads to a reduction of the ordering temperature, above which magnetization vanishes. This results in a shift of *M*_*s*_ vs. *T* curves as a function of moment dilution, thus increasing the relative change of magnetization with temperatures between 300 K and 575 K. Therefore, reducing moment dilution is effective both to reduce the variation of *M*_*s*_ with temperature and to increase the maximum operating temperature. Moreover, by combining *M*_*s*_ and *H*_*k*_ measurements at various temperatures, we derive the interfacial anisotropy energy per unit area *K*_*i*_, and show that it follows a power law dependence on *M*_*s*_, in good agreement with a previous report^17^. This simple relationship allows us to derive *H*_*k*_ for blanket films and Δ for patterned devices, over an extensive temperature range. To validate our approach, we compare the temperature dependence of Δ derived from full film data with direct measurements of devices integrated on complementary metal oxide semiconductor (CMOS) test chips. Extrapolated values are in remarkable agreement with measured data, showing the potential of our method to facilitate the design of thermally robust STT-MRAM film stacks.

## Results

**Figure 1:**
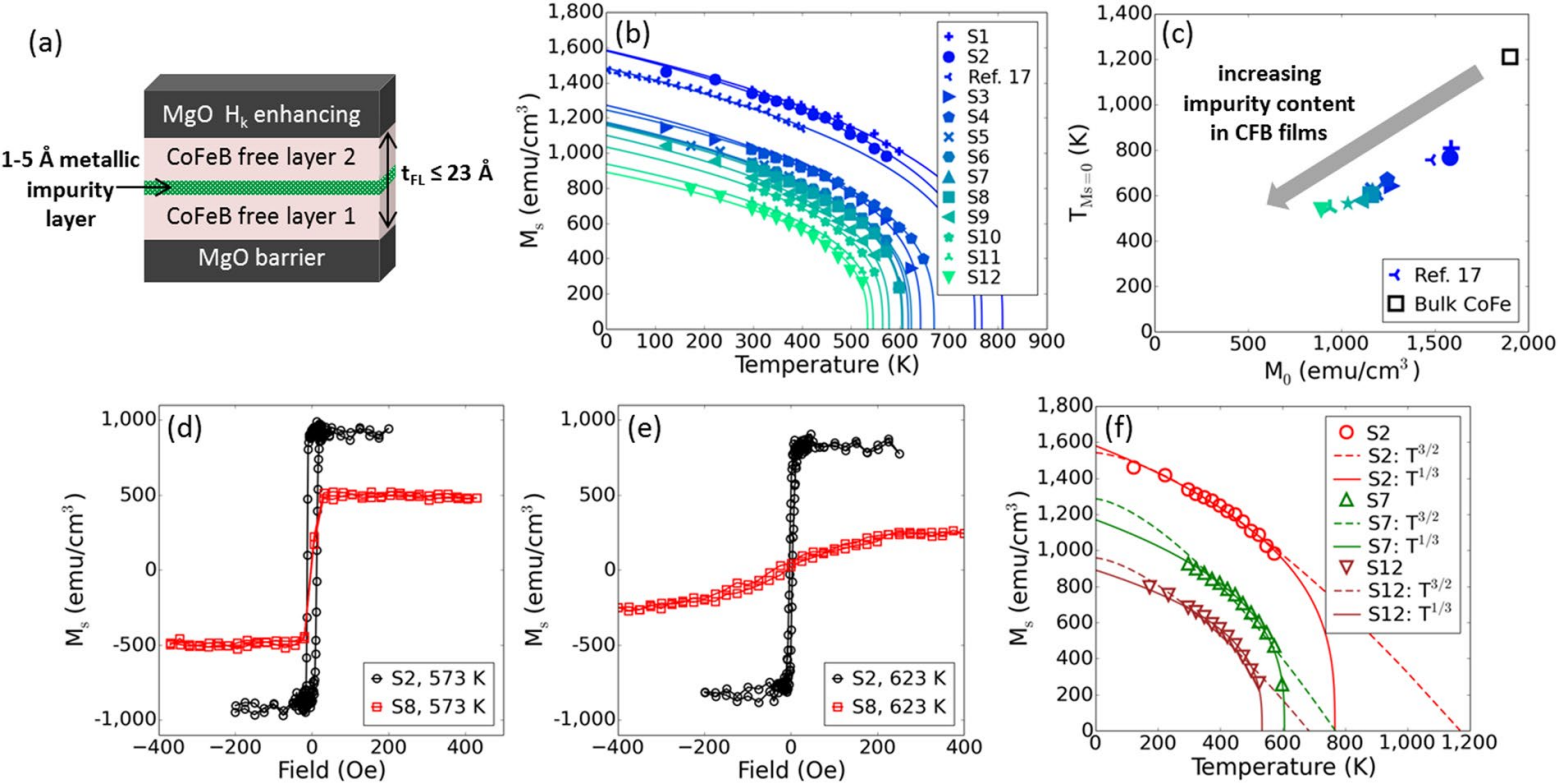
Magnetic properties of CFB free layers with different non-magnetic, metallic impurities for temperatures up to 650 K. (**a**) Schematic depicting CFB free layer with metallic impurity insertion. Layer thicknesses were not drawn to scale. (**b**) *M*_*s*_ for CFB free layers as a function of temperature. (**c**) *T*_*Ms*=*0*_ dependence on *M*_*0*_ for CFB free layers and a bulk CoFe alloy^31^. *M*_*0*_ and *T*_*Ms*=*0*_ were estimated using the *T*^1/3^ power law. (**d**) Minor loops of S2 and S8 measured at 573 K. (**e**) Minor loops of S2 and S8 measured at 623 K. (**f**) Comparison of *T*^3/2^ and *T*^1/3^ power law fits for the *M*_*s*_ dependence on temperature for select free layers.

We report on full MTJ film stacks, including seed layer, synthetic antiferromagnet reference layer, MgO barrier, CFB free layer, MgO *H*_*k*_-enhancing layer, and Ru/Ta-based cap. As depicted in Fig. 1(a), the nominal free layer thickness *t*_*FL*_ extends up to 23 Å and consists of Fe-rich (CoFe)_1-*y*_B_*y*_, where the Co:Fe ratio is at least 1:3 and *y* is 20–26 percent, with a thin non-magnetic, metallic impurity layer. The impurity layer is a group V-A or VI-A element such as Mo, W, or Ta with nominal thicknesses between 1 to 5 Å. All samples were annealed at 400 °C for 2.5 hours after fabrication. We use vibrating sample magnetometry (VSM) to measure the out-of-plane saturation magnetization *M*_*s*_ of the free layer for temperatures between −150 °C and 375 °C. Data for twelve samples labeled S1 to S12 with different boron and metallic impurity materials and concentrations incrementally increasing from 20 to 40 atomic % are shown in Fig. 1(b), along with data reprinted from a published low temperature study^17^. The value of *M*_*s*_ at room temperature decreases from 1350 to 680 emu/cm^3^ as the concentration of impurities increases. Most importantly, this reduction of *M*_*s*_ is also correlated with a reduction of the temperature at which magnetization vanishes *T*_*Ms*=0_ as shown in Fig. 1(c). The combination of reduced *M*_*s*_ and *T*_*Ms*=0_ results in a shift of *M*_*s*_ vs. *T* curves as a function of moment dilution, thus increasing the relative change of magnetization with temperatures between 300 K and 575 K. This suggests that moment dilution plays a key role in the high temperature behavior of CFB free layers for STT-MRAM, since it influences both the rate of variation of *M*_*s*_ with temperature and the maximum operating temperature. Hysteresis loops for high and low-*M*_*s*_ film stacks (Fig. 1(d,e)) measured near 600 K also confirm this conclusion: high-*M*_*s*_ film stack S2 retains much higher PMA than lower-*M*_*s*_ film stack S8.

The temperature dependence of *M*_*s*_ is fitted to a *T*^1/3^ power law^18^: ${M}_{s}(T)={M}_{0}\times {(1-\frac{T}{{T}_{Ms=0}})}^{1/3}$. Such a dependence, which has been reported for other ferromagnets^19^, is in principle valid only close to the Curie temperature where 0.88 < *T*/*T*_*Curie*_/0.988^18^. However, as shown by the solid lines in Fig. 1(b), all experimental data are well described by this relationship down to 200 K. Note that the temperature dependence of the magnetization at low temperatures follows the Bloch Law (~*T*^3/2^), which derives from magnon excitations that dominate at very low temperatures^17,20^. However this law is less suited to describe the high temperature regime relevant to STT-MRAM applications as shown in Fig. 1(f).

**Figure 2:**
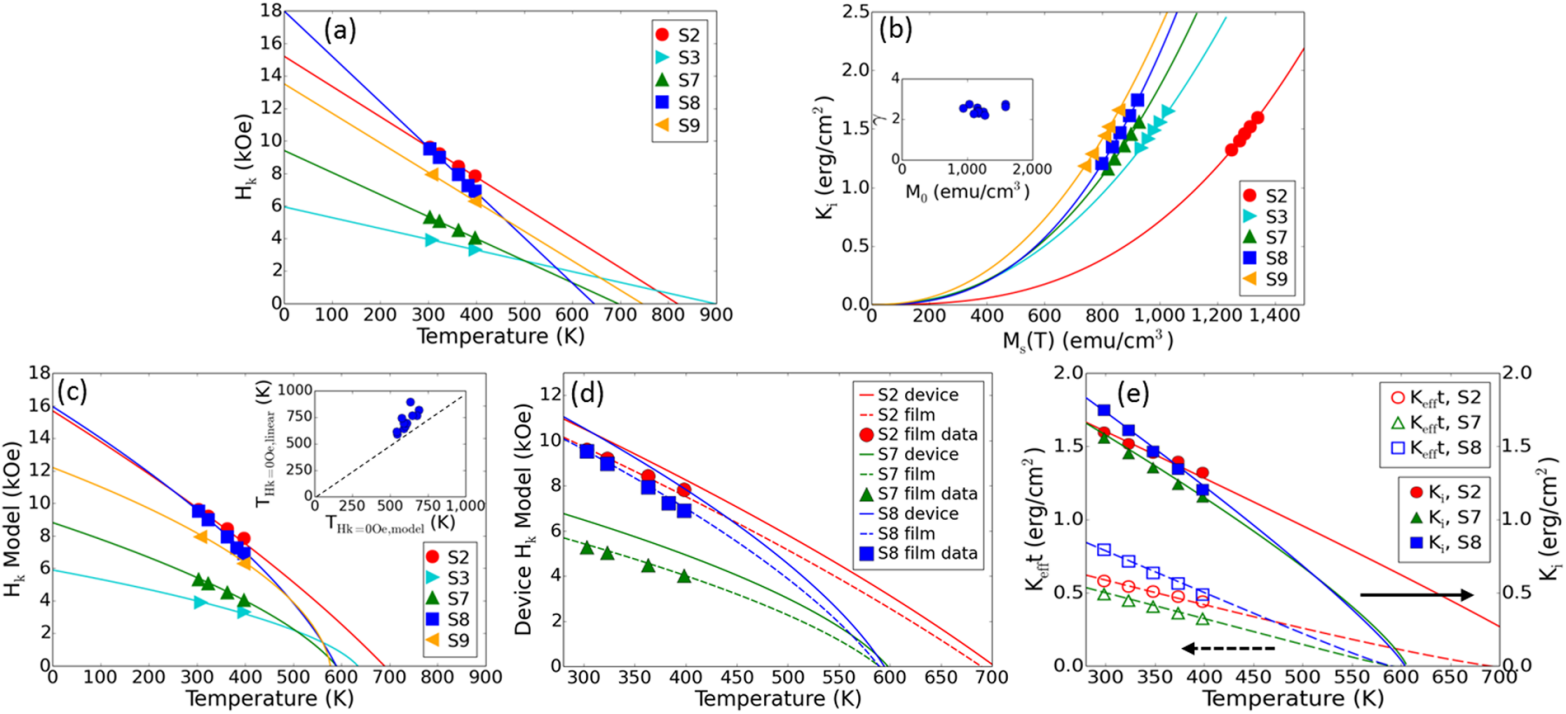
Anisotropy field and anisotropy constants of CFB free layers with different non-magnetic impurities. (**a**) Solid symbols show *H*_*k*_ measured by FMR between 300 to 400 K for five different film stacks. Solid lines show linear fits to the data. (**b**) Symbols show *K*_*i*_ calculated from experimentally measured values of *M*_*s*_ and *H*_*k*_, and fitted using a power law dependence on *M*_*s*_(*T*)^γ^ (solid lines). The inset shows the exponent γ as a function of *M*_*0*_ for free layers with different impurity amounts. (**c**) *H*_*k*_ data shown in (**a**) are compared with the values calculated from the temperature variations of *M*_*s*_ and *K*_*i*_ over an extended function of temperature. The inset shows the discrepancy in temperatures at which *H*_*k*_ vanishes between a linear approximation and the calculation. (**d**) Comparison of *H*_*k*_ models for devices (solid lines) and full films (dashed lines) of select stacks. The device model assumed a circular 70 nm diameter device. (**e**) Comparison of *K*_*eff*_*t*_*FL*_ (open) and *K*_*i*_ (solid) as a function of temperature.

The anisotropy field *H*_*k*_ measured by ferromagnetic resonance (FMR) as a function of temperature for selected samples are depicted with solid symbols in Fig. 2(a). In the temperature range of the FMR setup (between 300 and 400 K), *H*_*k*_ decreases approximately linearly with increasing temperature, as shown by the solid lines in Fig. 2(a). The effective anisotropy constant *K*_*eff*_ and the interfacial energy constant *K*_*i*_ are derived from *M*_*s*_ and *H*_*k*_ measurements: ${K}_{eff}=\frac{{M}_{s}\times {H}_{k}}{2}=\frac{{K}_{i}}{{t}_{FL}}-2\pi {M}_{s}^{2}$. The relationship between *K*_*i*_ and *M*_*s*_ is shown by the solid symbols in Fig. 2(b). Note that these data points only cover the temperature range accessible to FMR measurements. Linear approximation was used to interpolate *H*_*k*_ values at the same temperatures as the VSM measurements. As shown by the solid lines in Fig. 2(b), experimental data are well described by a power law dependence on *M*_*s*_(*T*), where ${K}_{i}(T)={K}_{i}(0){(\frac{{M}_{s}(T)}{{M}_{0}})}^{{\rm{\gamma }}}$ for a wide range of *M*_*s*_(*T*). The exponent ɤ is between 2.2–2.8, independent of *M*_0_ for all samples measured in this study (Fig. 2(b) inset). Interestingly, these values of ɤ for CFB films capped with MgO layers are in excellent agreement with that of ref.^17^, for which a metallic Ta cap was directly deposited on CFB. Combined with the lack of dependence of ɤ with the nature and concentration of non-magnetic impurities, this agreement suggests the universality of this relationship between *K*_*i*_ and *M*_*s*_ for CFB layers.

Since our experimental results allow us to determine the functional forms of both *M*_*s*_(*T*) and *K*_*i*_(*M*_*s*_) over a wide range of temperatures, we can now use these expressions to estimate *K*_*i*_, *H*_*k*_, *E*_*b*_, and Δ beyond the range of temperatures accessible experimentally. The result of these extrapolations is shown in Fig. 2(c–e) for *H*_*k*_, *K*_*i*_ and *K*_*eff*_*t*_*FL*_, respectively. As discussed below, the latter quantity is proportional to *E*_*b*_ per unit surface area in the case of uniform magnetization reversal. These extrapolations lead to several useful observations. Firstly, they show in Fig. 2(c) that the variations of *H*_*k*_ with temperature deviate from a linear dependence at higher temperatures, when *M*_*s*_ decreases rapidly. As a consequence, PMA vanishes at temperatures lower than those derived from the linear approximation. The discrepancy between the linear approximation and the model is summarized in the inset of Fig. 2(c). Secondly and perhaps most importantly, the energy barrier at high temperature cannot be assessed from the anisotropy or the energy barrier at 300 K. Indeed, as shown in Fig. 2(c,e) samples S2 and S8 exhibit similar *H*_*k*_ and *K*_*i*_ values at 300 K, while *K*_*eff*_*t*_*FL*_ is significantly larger for S8 than for S2. However, the temperature dependence of S8 is much faster than that of S2. Therefore, *K*_*eff*_*t*_*FL*_ vanishes below 600 K for S8, whereas S2 retains non-zero PMA up to almost 700 K. This demonstrates that the temperature dependence of thermal stability of CFB-based MTJ films stacks is determined primarily by the value of *M*_*s*_. This is an important result for the design of STT-MRAM suitable for high temperature data retention, for example reflow soldering compatibility or automotive applications.

In the following, we discuss the usefulness of our approach to make accurate predictions of the data retention of MTJ devices patterned to technologically relevant diameters. For patterned devices, the expressions used above for blanket films must be corrected to account for the reduction of the demagnetizing factor. The demagnetizing factor for a flat cylinder of diameter *d* is given by ${N}_{b}=1-(\frac{2}{\pi })(\frac{p}{k})[K(k)-E(k)]$, where ${k}^{2}=\frac{1}{1+\frac{1}{4}{p}^{2}}$, $p=\frac{{t}_{FL}}{d}$, and *K*(*k*) and *E*(*k*) are the complete elliptic integrals of the first and second kind, respectively^21^. The anisotropy field of patterned devices is thus given by ${H}_{k}=\frac{2{K}_{i}}{{M}_{s}{t}_{FL}}-4\pi {N}_{b}{M}_{s}$. For sub-100 nm devices, we have shown that device-level *H*_*k*_ can be significantly larger than the corresponding film-level values^22^. The temperature dependence of *H*_*k*_ calculated for 70 nm diameter circular devices from film-level measurements of samples S2, S7 and S8 are shown in Fig. 2(d).

**Figure 3:**
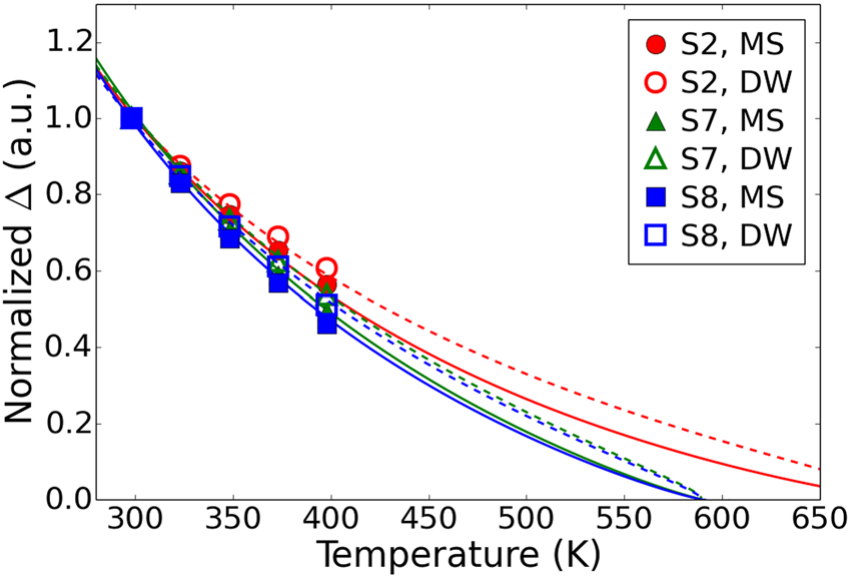
Temperature dependence of the normalized thermal stability factor. Values calculated for macrospin Δ_MS_ and domain-wall mediated Δ_DW_ reversal mechanisms are shown as solid and open, respectively, for different film stacks. High temperature extrapolations for the two mechanisms are depicted with the solid and dashed lines, respectively. All values are normalized to Δ_300 K_.

In order to compare the predictions of our model with actual data retention measurements, the thermal stability factor Δ must be calculated. The details of the calculation depend on the mechanism of the free layer’s magnetization reversal, which depends on the device diameter and magnetic properties. For devices smaller than approximately 30 nm in diameter, switching can be described by the macrospin approximation (MS), in which the free layer magnetic moment rotates uniformly. In this case, the energy barrier is given by E_*b,MS*_ = K_*eff*_St_*FL*_, where *S* is the device surface area^23^. For larger diameters, magnetization reversal is mediated by the nucleation and propagation of a domain wall (DW) across the device, leading to the following expression for the energy barrier^24^: ${{\rm{E}}}_{{\rm{b}},{\rm{DW}}} \sim d{t}_{FL}\sqrt{A\times {K}_{eff}}$. The expression also includes the exchange stiffness *A*. Since *A* also varies as ${M}_{s}^{2}$ ^25^, we can rewrite ${{\rm{E}}}_{{\rm{b}},{\rm{DW}}} \sim {M}_{s}d{t}_{FL}\sqrt{{K}_{eff}}$. In order to compare the relative change of Δ with temperature for these two magnetization reversal mechanisms for different free layer samples, Δ is normalized to the value at 300 K in Fig. 3. This calculation leads to two interesting findings. First, the relative change of Δ with temperature is nearly identical for both reversal mechanisms. Second, Δ exhibits a nonlinear dependence on temperature over the entire temperature range. This is an important finding for accurate extrapolations of Δ from experimental data retention measurements. Indeed, since thermal relaxation varies exponentially with Δ, small changes in temperature can lead to orders of magnitude changes in relaxation rate. Thus, direct measurements are only feasible in a fairly narrow temperature range^8^, typically a few tens of degrees, and extrapolations are needed to quantify Δ over the entire range of operating temperatures. Our results show that linear extrapolations over a wide temperature range lead to significant underestimation of data retention at those temperatures.

**Figure 4:**
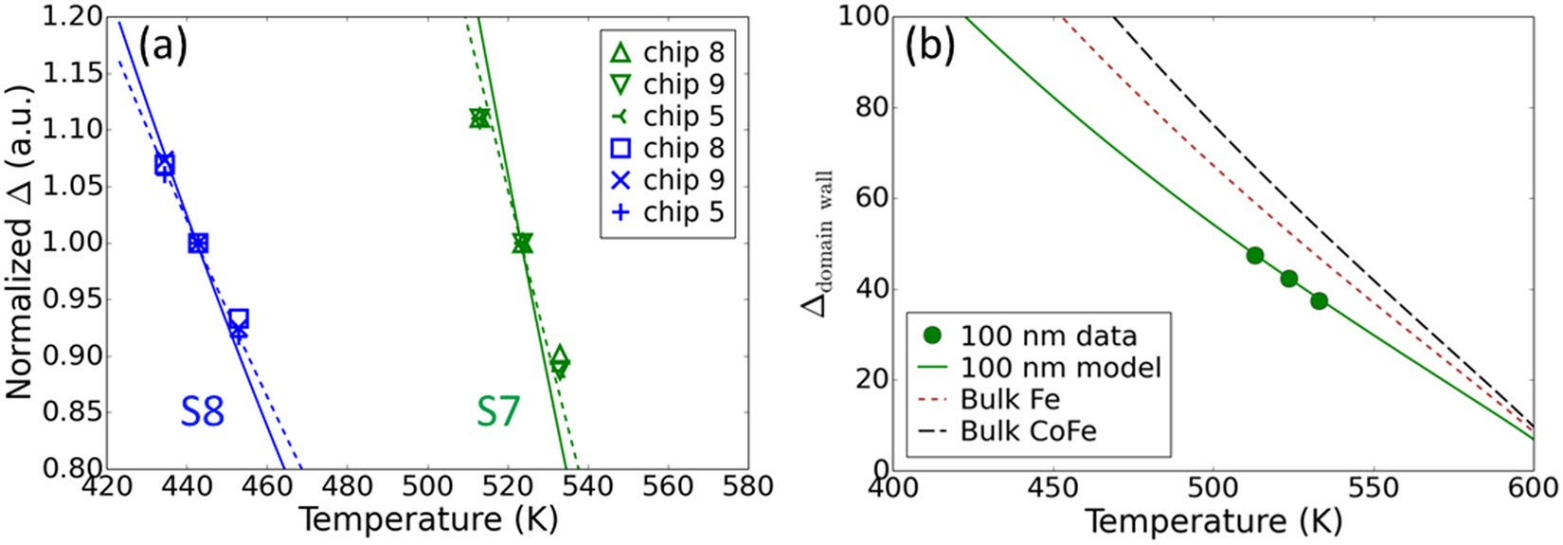
Comparison of Δ from chip data with calculated values. (**a**) Symbols show normalized values of Δ measured on integrated STT-MRAM chips for two different films stacks (S7 and S8) and three device sizes (chip 8, chip 9, and chip 5) with diameters ranging between 65 and 100 nm. Lines show extrapolations from film data Δ_MS_ (solid line) and Δ_DW_ (dashed line). (**b**) Experimental data for 100 nm diameter devices from film stack S7 () are compared with extrapolations for domain-wall mediated reversal calculated for bulk Fe and CoFe exchange stiffness (dotted and dashed lines, respectively), or by adjusting the exchange stiffness to fit the data (solid line).

We can now compare the results of our calculations directly with the values of Δ measured on actual STT-MRAM devices. These data are obtained by measuring the number of devices whose magnetization reverses as a function of the length of time the chips are baked at elevated temperature. We use fully functional 8 Mb chips integrated on CMOS circuits allowing us to probe error rates as small as a few parts per million. At such a deep error rate, we have shown that data retention is described by an effective thermal stability factor Δ_eff_, which encompasses both the median and standard deviation of the distribution of Δ^8^. Even though this method enables faster and more accurate measurements of data retention, as discussed above, practical limitations in bake time restricts the accessible temperature range. Data measured at three temperatures over a 20 K range for chips with MTJ stacks S7 and S8 are shown in Fig. 4(a). We have measured three different chips having device diameters ranging between 65 and 100 nm. Data are normalized to the value at the intermediate temperature for clarity. Solid and dashed lines show the results of the film-based calculations for MS and DW mechanisms, respectively. We find that calculations for both mechanisms give an accurate prediction of the relative change of Δ with temperature. The agreement is better for the DW reversal mechanism, as expected for the fairly large diameters of these devices.

Finally, we match the calculated value of Δ_DW_ with the experimental data to estimate the exchange stiffness constant *A* of the free layer. This comparison is shown in Fig. 4(b) for 100 nm diameter devices with MTJ stack S7. As discussed above, *A* is proportional to ${M}_{s}^{2}$ such that $A={A}_{0}{(\frac{{M}_{s}}{{M}_{0}})}^{2}$, where *A*_0_ is the exchange stiffness constant at 0 K. Experimental data are well fitted for *A*_0_ = 6.5 × 10^−7^ erg/cm. In order to compare this result with values for bulk Fe and CoFe, we use the following expression: $A=\frac{D{\rho }_{a}{\mu }_{a}}{2g{\mu }_{B}}$, where *D* is the spin wave stiffness, ρ_a_ is the atomic density, μ_a_ is the atomic magnetic moment, *g* is the g-factor, and μ_B_ is the Bohr magneton^26^. By using parameters for bulk Fe and CoFe from literature as summarized in Table 2, we find *A*_0_ = 22.7 × 10^−7^ and 35.8 × 10^−7^ erg/cm for Fe and CoFe, respectively. The sizeable reduction of *A*_0_ in sample S7 compared to bulk Fe and CoFe values is consistent with the dilution of moment due to boron and other non-magnetic impurities. Damage induced by nanofabrication processes may also contribute to reduced exchange stiffness^22^.

**Table 2:** Material parameters used for the exchange stiffness constant of bulk, body-centered cubic (BCC) Fe and CoFe.

	BCC Fe	BCC CoFe	Source
μ_a_ (μ_B_)	2.22 μ_B_	2.45 μ_B_	^27^
M_0_ (emu/cm^3^)	1757	1946	
a (Å)	2.861	2.858	^27^
ρ_a_ (atom/cm^3^)	8.54 × 10^22^	8.57 × 10^22^	
D (erg·cm^2^)	5.29 × 10^−29^ (330 meV·Å^2^)	7.53 × 10^−29^ (470 meV·Å^2^)	^28– 30^
g	2.21	2.21	
A_0_ (erg/cm)	22.7 × 10^−7^	35.8 × 10^−7^	

In conclusion, we have demonstrated the modulation of *M*_*s*_ and *T*_*Ms*=0_ by diluting the moment of CFB free layers with non-magnetic, metallic impurities. We find that *M*_*s*_ follows a *T*^1/3^ power law over a wide temperature range, and that the interfacial anisotropy *K*_*i*_ varies with *M*_*s*_^2.5±0.3^, independent of the material or concentration of impurities. These findings allow us to develop a simple model to extrapolate the temperature dependence of the thermal stability factor Δ over a wider range of temperatures than accessible experimentally. Extrapolations using this model are in excellent agreement with data retention measurements on integrated STT-MRAM chips. Our results show that the temperature dependence of Δ, which is detrimental to the energy efficiency of STT-MRAM, is primarily dependent on the free layer’s magnetization. Furthermore, our work gives a simple yet powerful method of improving the thermal design of STT-MRAM film stacks.

## Methods

All MTJ film stacks presented in this work were prepared using magnetron sputtering in an Anelva C-7100 deposition system at room temperature. After deposition, the blanket film wafers were annealed at 400 °C for 2.5 hours. For chip-level tests, circular devices with diameters between 65 to 100 nm were integrated into 8 Mb array CMOS wafers and patterned with UV photolithography and etched by reactive ion etching and argon ion beam etching. At the completion of the fabrication process, patterned devices were annealed at 400 °C for 2.5 hours. Vibrating sample magnetometry was used to measure the out-of-plane magnetic moment for temperatures ranging from −150 °C and 375 °C. *M*_*s*_ is defined as the magnetic moment normalized by the nominal free layer thickness. Ferromagnetic resonance spectroscopy was used to measure *H*_*k*_ for temperatures between 30 °C and 125 °C. Data retention measurements used temperature acceleration to predict data retention over the lifetime of the devices. Measurements were performed at elevated temperatures corresponding to an error rate between 10^−5^ to 10^−3^ following the procedure described in ref.^8^.

## Data Availability

The datasets generated during and/or analysed during the current study are available from the corresponding author on reasonable request.
